# Novel Strategies for Drug Discovery Based on Intrinsically Disordered Proteins (IDPs)

**DOI:** 10.3390/ijms12053205

**Published:** 2011-05-17

**Authors:** Jihua Wang, Zanxia Cao, Liling Zhao, Shuqiang Li

**Affiliations:** 1 Shandong Provincial Key Laboratory of Functional Macromolecular Biophysics, Dezhou 253023, China; E-Mails: qiayilai@mail.ustc.edu.cn (Z.C.); zll2008@mail.ustc.edu.cn (L.Z.); 2 Department of Physics, Dezhou University, Dezhou 253023, China; 3 Dana-Farber Cancer Institute, Harvard Medical School, Boston, MA 02115, USA; E-Mail: shuqiang_li@dfci.harvard.edu

**Keywords:** intrinsically disordered proteins, sequence characterizations, structural characterizations, interaction networks, drug-discovery

## Abstract

Intrinsically disordered proteins (IDPs) are proteins that usually do not adopt well-defined native structures when isolated in solution under physiological conditions. Numerous IDPs have close relationships with human diseases such as tumor, Parkinson disease, Alzheimer disease, diabetes, and so on. These disease-associated IDPs commonly play principal roles in the disease-associated protein-protein interaction networks. Most of them in the disease datasets have more interactants and hence the size of the disease-associated IDPs interaction network is simultaneously increased. For example, the tumor suppressor protein p53 is an intrinsically disordered protein and also a hub protein in the p53 interaction network; α-synuclein, an intrinsically disordered protein involved in Parkinson diseases, is also a hub of the protein network. The disease-associated IDPs may provide potential targets for drugs modulating protein-protein interaction networks. Therefore, novel strategies for drug discovery based on IDPs are in the ascendant. It is dependent on the features of IDPs to develop the novel strategies. It is found out that IDPs have unique structural features such as high flexibility and random coil-like conformations which enable them to participate in both the “one to many” and “many to one” interaction. Accordingly, in order to promote novel strategies for drug discovery, it is essential that more and more features of IDPs are revealed by experimental and computing methods.

## 1. Introduction

According to the traditional sequence-to-structure-to-function paradigm, active proteins have well-defined three-dimensional structures under physiological conditions. However, as early as in the 1990s, it was reported that there is another class of proteins, which have no well-defined structures under physiological conditions, but still have biological functions [1,2]. These proteins with intrinsically disordered regions (IDRs) are called intrinsically disordered proteins (IDPs). IDRs are defined as the regions that do not adopt any well-defined three-dimension structures under physiological conditions. IDPs are also called natively unfolded [2], natively denatured [3], and intrinsically unstructured proteins [4]. It has been established that IDPs lack stable tertiary or secondary structures under physiological conditions *in vitro*, they have high flexibility and random coil-like conformation, and they are highly abundant in nature [3]. In fact, it has been predicted that more than 30% of all eukaryotic proteins belong to this type [3]. The sequences of IDPs have low hydrophobicity, and they are substantially enriched in polar (such as R, G, Q, S, P, E, and K) and structure-breaking (such as G and P), disorder-promoting amino acid residues [3]. IDPs have unique structural features that enable them to participate in both one to many and many to one signaling, and they also have unique biophysical advantages, such as accessibility and space efficiency. IDPs are usually hub proteins in protein and other molecules interaction networks [5]. Recently, Uversky [6] concluded that the conformational behavior of IDPs is low cooperative.

IDPs play crucial roles in regulation, recognition, signaling, and control of protein-protein interaction networks. The importance of IDPs in biological functions is now being recognized. Some important endocytic proteins lack a tertiary structure and they play an important role in the field of endocytosis [7]. The functional repertoire of IDPs complements the functions of ordered proteins. They have close relationships with human diseases, such as tumor, amyloidoses, neurodegenerative diseases, cardiovascular disease and diabetes [5].

IDPs have brought great challenges to the classical structural and functional relationship of proteins, which is also a new opportunity to reveal the essential relationship especially for these types of disordered proteins. At the same time, intrinsic disorder is very common in disease-associated proteins. Drug-discovery has been linked with functions of IDRs, and new drug discovery strategies are aimed at a wide variety of signaling and regulatory functions. Therefore, the IDPs could serve as potential targets for structure-based drug design based on their prime features. Furthermore, IDPs are usually hub proteins in protein-protein interaction networks, and protein-protein interaction is a potential source for drug targets. In order to make full use of IDPs for drug discovery, it is important to study the characters of sequence, structure, dynamics, biophysics and protein-protein interaction network of these proteins. In this paper, we review characteristics of IDPs and highlight the novel strategies for drug discovery based on the updated research of IDPs.

## 2. Sequence Characterizations of IDPs

The biologically active conformation and three-dimensional structures of ordered proteins are determined by the amino acid sequences [8]. It could be deduced that the amino acid sequence also predominates the absence of ordered structures. IDPs have no well-defined structures under physiological conditions, which may come from its sequence characters. Thus more and more studies are focused on the sequence characters of IDPs to find the common features that encode the intrinsically disordered structures.

By analysis of amino acid composition of ordered and disordered segments, Dunker *et al.* [9] divided the 20 amino acids into three groups. The first group is called order-promoting amino acid (namely C, W, Y, I, F, V and L) which is depleted in disordered segments. The second group is called disorder-promoting amino acid (namely M, K, R, S, Q, P and E) which is enriched in disordered segments, and the third group includes A, G, H, T, N and D, which have no obvious differences between ordered and disordered segments and are thus called neutral residues. As some proteins with similar well-defined ordered structures have no obvious sequence comparability, there are also exceptions in the aforementioned class. The *N*-terminal (1∼39) of p53 (p53_1–39_) has been proved to be intrinsically disordered [10], but the content of leucine is up to 15.4% [11], which is thought to be an order-promoting amino acid and the statistical frequency value in disordered proteins is 5.44% [12], Leucine plays an essential role in the formation of hydrophobic cluster and the leucine-rich hydrophobic cluster promotes the formation of a collapsed structure of p53_1–39_ [11].

Compared with ordered proteins, IDPs have a lower sequence complexity [13], a larger net charge and a lower content of hydrophobic amino acid residues [14]. Lower mean hydrophobicity can restrain the formation of hydrophobic cluster and higher net charge can help to form an extended conformation by electrostatic repulsion. Uversky *et al.* [15] estimated the boundary between ordered and intrinsically disordered protein segments with mean net charge and mean hydrophobicity value as reaction coordinates. However, protein Rv3221c is special, it is proved to be intrinsically disordered, but its amino acid composition reveals that it has 24% charged and 54.9% hydrophobic amino acid residues and it adopts structured conformation at high temperatures [16].

Another issue of great interest to researchers is the prediction of IDPs. With some common features of amino acids, it is easy to predetermine whether proteins with certain amino acid compositions are IDPs. Disordered protein prediction has been one part of the Critical Assessment of Structure Prediction (CASP) from the 5th term [17], which is useful for inspiring rapid development. More than twenty predictors have been discussed based on the amino acid composition of the protein sequence [18] and physicochemical property of amino acids including aromaticity, net charge, flexibility, and hydropathy [13]. DISOPRED [19] chooses 21 parameters per residue as input and metaPrDOS [20] takes meta approach integrating the results of seven different prediction methods to predicate disordered regions. Using Bayesian multinomial classifier, the predictive accuracy of 89.2% could be achieved for intrinsically disordered regions [21]. Although the current predictors have higher predictive accuracy [22], it is notable that there is a deficiency for the predictors on intrinsic disordered region. For example, the residues 1–172 region of SKIP was demonstrated to be an intrinsic disordered region (SKIP172) [23], but was not predicted by neural network method [24]. In order to improve the accuracy, it is necessary for us to further study the characters of IDPs sequence and others. Furthermore, the prediction of protein binding regions in IDPs is very important, ANCHOR [25,26] is an original method for the prediction of disordered binding regions that are disordered in isolation but can undergo disorder-to-order transition upon binding.

## 3. Structural Characterizations of IDPs

Investigations of the structural characteristics of IDPs are the basis to reveal the molecular mechanism of their biological functions. Compared with structured proteins, IDPs are enriched in M, K, R, S, Q, P, E residues and possess a highly flexible, malleable random coil-like structure. The increased level of these residues makes them fail to fold into a fixed three-dimensional structure under physiological conditions [27]. Moreover, IDPs are composed of an ensemble of highly heterogeneous conformations.

At the structural level, Dunker *et al.* [3] had an alternative proposal called “The protein trinity”, which proposed that intrinsically disordered regions may exist in molten globule-like and random coil-like forms. Additionally, the pre-molten globule has been proposed as another form of disorder state by Uversky *et al*. with “The protein quartet” model [2]. The research results show that some residual structures have been confirmed in IDPs and they exhibit a rich diversity of local and even long-range structural preferences [28] as either coil-like or premolten-globule-like proteins [2].

The disorder of IDPs is crucial to their functions. The functions are considered to arise from any one of the three states or from transitions between disordered and ordered conformations [29]. The conformational changes associated with functions may be originated from alterations in environmental or cellular conditions.

DisProt [30] is a database which provides structural and functional information of IDPs. Our group has been trying to construct a second database of IDPs and IDRs in order to provide latest information on sequence, structural, biophysical and functional characterizations of IDPs and IDRs.

Human α-synuclein is a 140 amino acid-protein and its normal function is to bind to the surface of synaptic vesicles [31,32]. The oligomers of this protein have been linked to Parkinson’s disease and Lewy bodies. Typical of such ailments is the presence of α-synuclein aggregates in a β-structure that can be soluble or insoluble [33]. α-Synuclein protein is highly disordered when isolated in solution. The structure (PDB ID: 1xq8) of micelle-bound human α-synuclein has been discussed by Ulmer *et al*. [34] and shown as a partially helical structure. More detailed studies showed that this structure could take the form of curved α-helices with a break in the 38–44 regions on the micelle surface [34–36]. On the contrary, an uninterrupted helix has been proposed when α-synuclein is bound to lipid vesicles [37–40]. In addition, the protein can apparently interchange between the curved-helix and extended-helix conformations, in the presence of small spheroidal detergent micelles, the extended-helix conformation can convert into a curved-helix. Membrane-bound conformations of α-synuclein likely mediate the protein’s function and play a role in the aggregation and toxicity of the protein. Recently, Georgieva *et al*. [41] studied the influence of different environments on structural character of α-synuclein, which concluded that: when α-synuclein is free in solution or in the absence of membrane or detergent, the conformation fluctuates between compact and extended; when α-synuclein is in the detergent, the conformation selected is dependent on the detergent concentration; the membrane environment exerted an influence on the conformation of α-synuclein protein. The ability of several polyphenols (exifone) to inhibit the assembly of α-synuclein was investigated by many researchers. Yamaguchi *et al*. [42] studied the characterization of exifone treated α-synuclein monomer and dimmer, their results showed *N*-terminal region (1–60) is involved in the inhibitor-induced dimerization. De Genst *et al.* [43] studied the structure and properties of a complex of α-synuclein and an antibody NbSyn2, and they found that NbSyn2 binds specifically to monomeric α-synuclein and interacts with four *C*-terminal residues of α-synuclein.

The absence of well-defined structure for IDPs under the physiological condition without partner makes it impossible to obtain a unique high-resolution structure. The structural studies on IDPs are to gain some characteristic parameters (customarily experimental data) of ensemble states that are sampled by polypeptide.

Many experimental techniques applied to ordered proteins could also be applied to IDPs. With x-ray crystallography method, disorder results in missing electron density in determined structures. The region without coordinates of residues atoms in crystal structure is determined as intrinsically disordered [3]. Nuclear magnetic resonance (NMR) spectroscopy is a powerful technique for protein structure determination and dynamics characterization in solution. With the ^15^N–^1^H heteronuclear nuclear overhauser effect (NOE) measurement, ordered residues hold positive values and disordered residues hold negative values [44,45]. Based on the NOE data, a series of consecutive positive value means ordered region and a series of consecutive negative value means disordered region. Paramagnetic relaxation enhancement (PRE) has perhaps been the most successful at detecting long-range contacts in disordered protein ensembles [46,47]. In recent years, several other techniques have been used to identify IDPs, such as: residual dipolar couplings (RDCs) steady-state fluorescence spectroscopy [48], circular dichroism (CD), differential scanning calorimetry (DSC), surface plasmon resonance (SPR), electrospray mass spectrometry [49], Fourier transform infrared (FTIR) [16], Raman spectroscopy and Raman optical activity [50]. In general, more than one method are used synchronously [23,51–54].

In addition, computational methods increasingly play important roles in depicting disordered protein structures and dynamics behaviors [5]. It can generate *de novo* prediction [11] and experimental-data-based prediction [54]. With experimental data as ensemble average, computational simulation could study conformational characters in detail [55]. With computation simulations, p53_1–39_ shows bimodal behavior [11] which suggests the coexistence of both ordered and disordered structure in solution. Bimodal behavior has been suggested to be an intrinsic characterization of IDPs in solution [56]. Studies on IDPs have indicated that polar IDPs prefer ensembles of collapsed structures in aqueous solutions [50,57], and p53_1–39_ has been observed to collapse more quickly than others with an average collapsing time of 52ns starting from extended conformation [11]. Cao *et al*. [58,59] investigated the structural and thermodynamics characters of α-syn12 peptide (residues 1–12 of the human α-synuclein protein) in aqueous solution, and they showed that the isolated α-syn12 peptide in water adopted four different conformational states.

Although IDPs are similar in many aspects to proteins that are unfolded due to denaturation, they have striking differences [60]. The former tend to cluster in the mainly disordered/irregular region of the non-linear mapping (NLM) plot and appear to contain a significant amount of the extended PPII-helical conformation; whereas the latter appear in other regions and can contain significant amount of β-structure in the case of reduced proteins and α-helix in the case of acid molten globules [61]. Proline, a disordered-promoting amino acid, is known to disfavor a rigid secondary structure but has a strong preference for the left-handed polyproline II (PP II) helix [62] and is the poorest β-strand-forming residue [61].

## 4. IDPs Interaction Networks

### 4.1. The Human Disease Network and Diseasome

Recently, a revolutionary concept named “the diseasome”, defined as a combined set of all known disorder/disease gene associations, offers a platform to explore, in a single graph-theoretic framework, all known phenotype and disease gene associations, thus indicating the common genetic origin of many diseases [63]. The human disease network is a set of all known human genetic diseases. In the human disease network (HDN) nodes represent disorders, and two disorders are connected to each other if they share at least one gene in which mutations are associated with both disorders. The diseasome is constructed based on the relationship between the human disease network and the disease gene network [63]. The diseasome provides rapid visual references of the genetic links between disorders and disease genes, indicating the possibility of discerning general patterns and principles of human diseases not readily apparent from the study of individual disorders. HDN reflects the underlying cellular network-based relationship between genes and functional models.

### 4.2. Unfoldome of Human Genetic Diseases

The human-genetic-diseases-associated unfoldome, which is defined as the IDP-containing subset of a given genome, is associated with human genetic diseases [63–65]. It is reported that intrinsic disorder is common in diseasome, and proteins from different diseases possess different levels of intrinsic disorder. Many disordered regions are subjected to alternative splicing and contain specific molecular recognition features responsible for the protein-protein interactions. Many hub proteins are generally more disordered than non-hub proteins.

### 4.3. IDPs in Human Protein-Protein Interactions

IDPs are *considered* to play an important biological role in protein-protein interactions and have shown to participate in both one-to-many and many-to-one signaling. In order to understand the role of a protein in any cellular mechanism, it is critical to identify its gene networks. For example, there are 147, 65 and 140 proteins involved in Huntington disease, Parkinson’s disease and Alzheimer’s disease, respectively [66]. Kana *et al*. [67] compared the frequency of different interactions in a human protein-protein interaction network and found that human protein-protein interactions preferably occur between disordered proteins and the flexibility of the interacting protein may play an important role in protein interaction networks. Swasti *et al*. [66] have investigated the content of unstructuredness in three neurodegenerative diseases datasets, and found significantly high prevalence of unstructured proteins in most of these diseases.

Proteins that have a large number of interactions are called hubs [68]. It is obvious that hubs are central to the normal functions of the protein-protein interaction network in every organism. Several studies showed that hub proteins are implicated in diseases. The structural flexibility of IDPs allows them to adopt different structural conformations when bound to different targets and affects their binding abilities [69]. For example, human α-synuclein presents partial folding with several divalent and trivalent mental ions [70], and it undergoes conformational change from an unstructured monomer in solution to organized structure when interacting with phospholipids [71]. This organized structure forms the basis of aggregation and fibrillation. Any factors which lead to population of this organized structure will increase the likelihood of α-synuclein fibril formation. Patil *et al*. [68] found proteins with a more diverse domain composition are over-represented in hubs compared with non-hubs.

## 5. Drug-Discovery Pathways Based on IDPs

### 5.1. IDPs Can Be Used to Design a Novel Inhibitor to Avoid Amyloid-Like Aggregation

More than 40 human diseases have been associated with the formation extracellular fibrilar aggregates [72] that are generally known as amyloid fibrils. IDPs have a higher net charge and lower hydrophobicity [2], and they also have a lower number of aggregating sequences, which IDPs use “classical” strategies to avoid amyloid aggregation, providing a novel pathway to design inhibitors to prevent amyloid-like aggregation [73].

### 5.2. Drug Design Based on Transition from Disordered to Ordered

The intrinsic disorder proteins could serve as potential targets for structure-based drug design which stress the transition from disordered to ordered confirmation through drug stimulation [74]. Two years ago, an unstructured domain of a regulatory protein was found to be involved in inhibiting catalytic activity of insulin signaling in the treatment of diabetes [75,76]. Based on the features of IDPs, they propose a hypothesis that disease associated proteins can be targeted for structural transition by using structure based drugs that mimic the binding partner of targeted IDPs and induce moderation in structure and behavior of the targeted IDP. Therefore it may be possible to alter the folding of target proteins to regulate its activity and ultimately its function.

### 5.3. Drug Discovery Based on IDPs Interaction Networks

Intrinsic disorder is very common in disease-associated proteins, giving rise to the disorder in disorders phenomena [68,77]. Protein-protein interaction is a potential source for drug targets [64]. Protein interactions and understanding of the results at a deeper level may predict the interesting drug targets. People have been trying to develop drug molecules that block protein-protein interactions.

Most hub proteins in interaction networks have intrinsically disordered regions. There is large local flexibility in partially disordered hub proteins and global flexibility in fully disordered hub proteins. Some hubs contain both ordered and disordered regions, and some hubs are entirely ordered [69]. For the highly structured hubs, the binding regions of their partner proteins are intrinsically disordered (ID) [78]. Intrinsic disorder is utilized in protein-protein interactions: namely, one disordered region binding to many partners and many disordered regions binding to one partner [78,79]. Many IDPs and IDRs fold upon binding with their specific partners. Protein conformational diseases may result from not only protein misfolding but also misidentification and mis-signaling [78].

Alternative splicing is commonly present in several genetic diseases. Alternative splicing regions in corresponding proteins are predicted to be highly disordered. Recently several small molecules as potential drugs have been shown to act by blocking protein-protein interactions based on intrinsic disorder of one of the partners [80]. Various disease-associated proteins are very rich in such disorder-based drug discovery targets. Although there has not been any drug molecule that functions by inhibiting a protein-protein interactions, several promising molecules are encouraging a renewed interest in this approach [81]. Several interesting drug-like lead compounds apparently function by blocking protein-protein interactions, and these leads are being actively pursued via drug-discovery strategies. The p53/Mdm2 interaction has been the focus of multiple drug-discovery studies, and the binding region of p53 is intrinsically disordered. Bioinformatics and computational biology tools were employed to reveal the features of disordered region for finding drug-discovery targets. By this approach, thousands of possible new drug targets involving one disordered partner were found. Lots of new drug targets were found for each of the major diseases [82]. The interaction between disordered and ordered proteins has several features that are consistent with being a good target for drug discovery. The interface between one structured and disordered partner is almost never flat. Such interaction is likely to be weaker than similar sized interaction between two structured proteins. Protein-protein interactions with disordered interface regions are as new targets for drug discovery. Development of tissue-specific drugs by taking into account tissue-specific alternative splicing in disordered regions from protein-protein interaction that can be blocked by small molecules.

Drug-discovery has been linked with the function of IDRs. New drug discovery strategies aim at targeting a wide variety of signaling and regulatory functions of these regions.

### 5.4. Examples of Drug Discovery Based on a Few IDPs

**α-synuclein** [64]: α-synuclein is a typical IDP that links various synucleinopathies. The structure of α-synuclein is extremely sensitive to the environment and can be easily modified. α-synuclein is an example of a disordered hub, and is shown to interact with at least 50 ligands and other proteins [83]. A recent proteomic analysis identified 587 proteins involved in the formation of complexes with α-synuclein in the dopaminergic cells, with 141 proteins displaying significant changes in their relative abundance after these cells were treated with rotenone [84].

The orphan G protein-coupled receptor 3(GPR3) modulates amyloid beta peptide generation in neurons, and it represents a potential therapeutic target for the treatment of Alzheimer’s disease [85].

**Tau** protein [86]: Efforts to develop drugs that halt the relentless brain degeneration caused by disorders have only met with modest success so far. The approved therapies only temporarily slow cognitive decline, possibly because they do not target the root cause of the disease. They are aimed at reducing production of amyloid beta, a protein fragment thought to be the instigator of the nerve cell death driving Alzheimer’s disease [87]. Recently, researches are taking a closer look at another possible target, a protein called tau that is involved in the pathology of a number of neurodegenerative diseases, including Alzheimer’s disease. Drugs, such as FTID, decreases tau phosphorylation and inhibit neurofibrillary degeneration.

**P53 protein** [64]: p53 is at the center of a large network, regulating expression of genes involved in numerous cellular processes including cell cycle progression, apoptosis induction, DNA repair, and response to cellular stress, *etc*. When p53 function is lost, cells often undergo cancerous transformation [88]. A database of p53 point mutations was created. There are three structural domains in p53 [89]: *N*-terminal translational activation domain, central DNA binding domain, and *C*-tramerization regulatory domain. DNA binding domain is intrinsically structured, whereas the two terminal domains are intrinsically disordered. P53 induces or inhibits over 150 genes. Overall, about 70% of the interactions between p53 and its partners are mediated by IDRs in p53 [78]. A bias towards intrinsic disorder is even more pronounced in the site of posttranslational modifications, with 86%, 90%, and 100% of observed acetylation, phosphorylation, and protein conjugation sites, respectively. p53 extensively utilizes IDRs to mediate and modulate interactions with other proteins.

Cancer is a disease of cell biology [90]. p53 network, in response to DNA damage, provides us a new edificatory to study IDPs-associated protein-protein interaction network [91], and IDPs-based research may provide novel pathways for cancer treatment.

### 5.5. The Key Roles of Computation in Drug Discovery

It is a fact that the use of computational methods has been involved in all aspects of drug discovery today [92]. With a computational tool, one can find more new drug candidates quickly at a lower cost. Currently, a revolutionary concept is computational unfoldomics, which is involved in IDPs discovery, predicting IDPs, computer simulations of IDPs, finding functional sites in IDPs, fuzziness of protein-protein interactions and finding order in disorder, and so on. It is obvious that computational unfoldomics will improve the IDPs research and promote drug discovery.

## 6. Conclusions

We should enlarge our view of what constitutes human disease research, recognizing that the discoveries that have the most profound impact on disease treatments emanate from basic research on model organisms, rather than from studies of highly complex human diseases. The studies on IDPs belong to these basic researches, which could provide us novel pathways for drug discovery. However, it is not enough for us to design new drugs based on the current understanding of the features of IDPs. Therefore it is crucial to further reveal novel features of IDPs, including sequence, structural, dynamics, biophysical and interaction network, by both computational and experimental methods. Drug discovery based on these novel features may reveal a bright new path.
